# Kinetic Modeling of Corn Fermentation with *S. cerevisiae* Using a Variable Temperature Strategy

**DOI:** 10.3390/bioengineering5020034

**Published:** 2018-04-24

**Authors:** Augusto C. M. Souza, Mohammad Mousaviraad, Kenneth O. M. Mapoka, Kurt A. Rosentrater

**Affiliations:** Agricultural and Biosystems Engineering Department, Iowa State University, Elings Hall, 605 Bissell Road, Ames, IA 50011, USA; acsouza@iastate.edu (A.C.M.S.); mousavi@iastate.edu (M.M.); komapoka@iastate.edu (K.O.M.M.)

**Keywords:** fermentation, *Saccharomyces cerevisiae*, kinetic modeling

## Abstract

While fermentation is usually done at a fixed temperature, in this study, the effect of having a controlled variable temperature was analyzed. A nonlinear system was used to model batch ethanol fermentation, using corn as substrate and the yeast *Saccharomyces cerevisiae*, at five different fixed and controlled variable temperatures. The lower temperatures presented higher ethanol yields but took a longer time to reach equilibrium. Higher temperatures had higher initial growth rates, but the decay of yeast cells was faster compared to the lower temperatures. However, in a controlled variable temperature model, the temperature decreased with time with the initial value of 40 ${}^{\circ }$C. When analyzing a time window of 60 h, the ethanol production increased 20% compared to the batch with the highest temperature; however, the yield was still 12% lower compared to the 20 ${}^{\circ }$C batch. When the 24 h’ simulation was analyzed, the controlled model had a higher ethanol concentration compared to both fixed temperature batches.

## 1. Introduction

Fermentation is a chemical process by which fungi or bacteria metabolize sugar to acids, gases, or alcohol in an environment with lack of oxygen. The two most common species used in this process are *Saccharomyces cerevisiae* and *Zymomonas mobilis*, where the first one is a fungus used more for industrial processes [1]. Several biomasses can be used for this purpose including grasses, wood, and commodity grains. For corn, the starch is the primary glucose source for fermentation. The most commonly used method to increase ethanol production involves grinding the corn to fine grades to increase surface area during fermentation.

Fuel ethanol and beverage alcohol are two examples of products that require fermentation to be produced. In the United States of America (USA), ethanol is commonly used domestically and produced mainly from corn. Since large quantities of ethanol are much needed in the USA, production of corn and lignocellulose must be high to meet demand. For 2016, the production of fuel ethanol was 366,981 thousand barrels, and consumption was of 341,817.322 thousand barrels [2]. According to the USDA [3], a total of 15,345 million bushels of corn were produced in the year of 2015/2016, where 1304 million bushels were destined to ethanol production, which means that 31.5% of total produced corn in the US was used for fuel, beverage, solvent, and other uses. For instance, around 23.153 million 9-L cases of Whiskey, a corn-based alcoholic beverage, were produced in the year of 2017, leading to revenue of $3.368 billion dollars for the American Whiskey suppliers [4].

Heat is also a byproduct of alcoholic fermentation, which can increase the temperature of the process. During ethanol fermentation, yeast is subject to several forms of stress, such as temperature changes, nutrient deficiency, and bacteria contamination [1]. In addition, ethanol accumulation inhibits yeast cell growth and consequently slows down ethanol production. All these forms of stress should be monitored due to its effect on yeast viability and vigor to optimize alcohol yield rate.

In ethanol and other fermentation outputs, several limiting factors can be altered to maximize and optimize by-products’ production during this process. A corn fermentation study to produce ammonium lactate (an essential component for ruminant feeds) was conducted by Mercier et al. [5]. The ammonium lactate is produced by neutralizing the lactic acid from corn fermentation. In this study, pH was controlled to improve ammonium lactate production and found that maximum rate was reached with pH between 6.0 and 6.5. Enzyme activity is also influent to the production of ethanol; therefore, an investigative model was developed to evaluate the enzyme activity. Altintas et al. [6] developed a kinetic model, which investigated the nonlinear enzymatic effect and how they are affected by heterologous enzymes. The authors concluded that xylose isomerase optimized ethanol production in a continuous fermentor. Moreover, Phisalaphong et al. [7] investigated the effect of temperature on kinetic parameters of ethanol fermentation using cane molasses as substrate. The results showed that fermentation kept at 30 ${}^{\circ }$C had higher ethanol concentrations than the models with the other higher temperatures studied (33, 35, 38, and 42 ${}^{\circ }$C).

Although the corn fermentation process is a well-established process, there is still space for improvement. Better monitoring of ethanol production can significantly improve the production of alcohol. Bialas et al. [8] investigated the effects of mash concentration, enzyme dose, and pH on saccharification and corn starch fermentation for ethanol production. In this study, the mash temperature was kept constant at 35 ${}^{\circ }$C; however, there was no report about how the temperature was monitored or controlled during the process. Öhgren et al. [9] studied two different process configurations. Simultaneous Saccharification and Fermentation (SSF) were used concurrently, with the temperature below 35 ${}^{\circ }$C, and Separate Hydrolysis and Fermentation (SHF), where the temperature of the fermentation was kept at 45 ${}^{\circ }$C for 120 h. For most cases, the results showed that SSF had higher ethanol yields compared to SHF; however, the temperature was said to be constant.

Creating models to observe the production of ethanol from biomass needs to be continually studied to investigate the numerous variables that can affect the commercialization of the final product. Indeed, mathematical software is necessary to calculate and analyze such models due to its high complexity. Based on computer modeling, the temperature could be controlled during the fermentation process to investigate the effect on ethanol yields.

The overall objective of this study was to implement a fermentation kinetic model to analyze/determine the amount of ethanol produced within a stipulated time, subjected to the catalytic microorganism (*Saccharomyces cerevisiae*). Additionally, this study aims to investigate the performance of the yeast at different fixed and controlled variable temperatures and determine how these affect the production of ethanol. The specific objectives were to simulate and analyze the fermentation process at different temperatures, perform a parametric sensitivity analysis as a goal to assess the effect of temperature, yeast concentration, and time on the production of ethanol and also design and implement a simulation model with changing temperature to optimize ethanol production.

## 2. Materials and Methods

As previously stated, in a medium rich with glucose, the yeast begins the process of fermentation under an anaerobic environment. However, the growing presence of alcohol inhibits its growth and consequently decreases the ethanol production rate in the batch.

The fermentation temperature and time should be considered when modeling the yeast behavior and ethanol production. In higher temperatures, yeast tends to grow and produce ethanol through fermentation faster compared to low temperatures. The same alcohol concentration promotes cell death, which will also be faster at higher temperatures, consequently leading to a quicker decay of living cells [10]. Thus, the ideal process is to achieve an optimum point of temperature for high ethanol production and steady cell mass growth rate.

### 2.1. Fermentation Modeling

Nanba et al. [10] proposed a model where the ethanol production and yeast growth are governed by a nonlinear system as observed in Equations (1) and (2). The system has two state variables: ethanol concentration in g/L (*p*) and cell mass concentration in g/L (x):

(1) $$\frac{dx}{dt}=\mu x,$$

(2) $$\frac{dp}{dt}=(\alpha \mu +\beta )x,$$

where μ is the specific cell growth rate (h${}^{-1}$), α is a growth-associated coefficient, and β(h${}^{-1}$) is the specific rate of non-growth associated ethanol production consisting of maintenance and energy uncoupling terms. The ethanol production rate (h${}^{-1}$) can be interpreted as

(3) v=(αμ+β).

Both parameters μ and β also change with time and ethanol concentration, whose initial values depend on some thermodynamic constants. These parameters can be described by the following equations:

(4) $$\mu =\mu_{0}\prod_{i=0}^{m-1}e^{-k^{\prime }p_{i}^{n}\Delta t},$$

(5) $$\beta =\beta_{0}\prod_{i=0}^{m-1}e^{-k^{\prime \prime }p_{i}^{n}\Delta t},$$

where $\mu_{0}$ and $\beta_{0}$ are the initial values for μ and β; $k^{\prime }$ and $k^{\prime \prime }$ are deactivation constants; *n* is the ethanol inhibition degree; $p_{i}$ is the ethanol concentration at discrete step *i* and Δt is the time step. The deactivation constants, $\mu_{0}$, and $\beta_{0}$ can be calculated according to the following Arrhenius equation since they are constants related to chemical reactions dependent on temperature:

(6) $$\mu_{0}=A_{1}e^{\left(-\frac{E_{1}}{RT}\right),}$$

(7) $$k^{\prime }=A_{2}e^{\left(-\frac{E_{2}}{RT}\right),}$$

(8) $$\beta_{0}=A_{3}e^{\left(-\frac{E_{3}}{RT}\right),}$$

(9) $$k^{\prime \prime }=A_{4}e^{\left(-\frac{E_{4}}{RT}\right).}$$

$A_{1}$, $A_{2}$, $A_{3}$, and $A_{4}$ are constants that depend on the frequency of activated complex formation from the reactants and E is the activation energy. For the reaction, these are all thermodynamics parameters that were determined empirically [10]. R is the gas constant and equals 1.987 cal K${}^{-1}$ mol${}^{-1}$, and *T* is the temperature in Kelvin. For lower temperatures, it can be observed that both initial cell growth rate and ethanol production rate will be lower compared to high temperatures. Based on experimental data, the values for *A* and *E* could be determined by nonlinear modeling as shown in Table 1.

Experimental data from different authors were used to validate the data from the computational model following the same characteristics from these studies [11,12].

### 2.2. Variable Temperature Control

Temperature has a significant influence on the production of ethanol. This can be concluded by directly looking at the Arrhenius equations. Higher temperatures have higher initial values for ethanol production rate and cell growth rate; on the other hand, higher rates mean that ethanol will be produced faster, and inhibition of cell growth will be more prominent, which can lead to smaller final ethanol concentration compared to lower fermentation temperatures.

Fermentation is a process that releases energy in the form of heat. If the temperature of the batch can be controlled with time, both ethanol production and cell growth can be optimized to have a faster and more productive batch fermentation.

The *Saccharomyces cerevisiae* is a mesophilic organism, that is, it has an optimum growth temperature between 28 and 35 ${}^{\circ }$C [13]. Kishimoto also showed how this yeast has a higher specific growth rate in a fermentation process at 35 ${}^{\circ }$C than at 28 ${}^{\circ }$C. However, the final ethanol yield was smaller for the higher temperature compared to the lower one. Based on that, a fermentation model was designed where the temperature was controlled over time. The temperature was initiated at 40 ${}^{\circ }$C, to take advantage of the higher specific growth, and exponentially reduced to a final temperature of 20 ${}^{\circ }$C to optimize the final ethanol yield. The chosen temperature behavior was in the form of Equation (10):

(10) $$T\left(t\right)=T_{0}e^{-ct},$$

where *c* is a constant, derived from Equation (11):

(11) $$c=\frac{-\ln\left[\frac{T(t)}{T_{0}}\right]}{t}.$$

Considering that the initial and final value of temperature were, respectively, 40 ${}^{\circ }$C and 20 ${}^{\circ }$C, and *t* is the total time of the fermentation, *c* will be equal to 0.0116 h${}^{-1}$ for a simulation of 60 h and 0.0289 for a 24 h simulation; thus, Equation (10) will have the following form:

(12) $$T\left(t\right)=40\times e^{-ct}.$$

For this simulation, not only were the ethanol and yeast concentrations variables, but also the temperature. Thus, every time the temperature changed, a new set of kinetic parameters had to be recalculated.

This study did not take into consideration contamination of bacteria or another organism in the batch. It was assumed that the pH was constant and under control the entire time. In addition, the concentration of glucose in the batch was considered sufficient, minimizing competition for nutrients during the fermentation.

### 2.3. Sensitivity Analysis

In this project, a parametric sensitivity analysis was also performed to investigate the model’s performance. To execute the sensitivity analysis, a series of simulations were required. The Latin Hypercube Sampling (LHS) method was chosen to produce design points for the necessary simulations. LHS is a space-filling design method, which maximizes the minimum distance between design points and requires even spacing of the levels for each factor [14]. This method produces designs that mimic the uniform distribution of design points over parameters’ domain. Yeast concentration, temperature, and fermentation time were the deterministic parameters of interest for the sensitivity investigation over final ethanol concentration and ethanol production rate (final production divided by fermentation time) as response parameters. The range of values for the parameters was decided based on optimal range reported in the literature with some expansion. Table 2 shows the range of values for variable parameters.

After implementing the design points and running the model to predict response values, a Gaussian process model was used to investigate the sensitivity of input parameters on response variables. The Gaussian Process platform fits a spatial correlation model to the data. The correlation of the response between two observations decreases as the values of the independent variables become more distant. This platform was used to quantify the main and marginal effects, in addition to the total sensitivity of each factor. The JMP [15] software package (JMP^®^ Pro 13.2.0, SAS Institute Inc., Cary, NC, USA) was used to produce the design points and perform sensitivity analysis.

## 3. Results and Discussion

### 3.1. Constant Temperature Modeling

**Figure 1:**
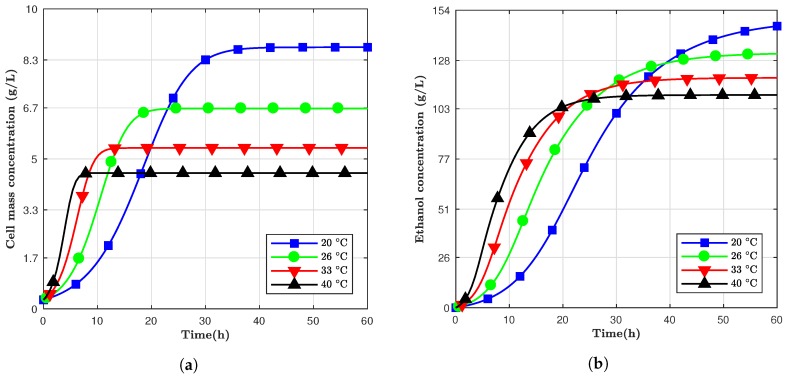
Simulation results using fixed temperature for (**a**) cell mass concentration; (**b**) ethanol concentration.

The results of the simulation with four different values for temperature are shown in Figure 1. As previously described, for this scenario, the temperature was fixed for the entire simulation of the process.

The models developed to predict ethanol are all nonlinear, and they depend mostly on variables such as time and temperature in which those two factors naturally influence the cell mass and ethanol concentrations. Figure 1 above shows the morphology of ethanol in a batch based on different temperatures (20 ${}^{\circ }$C to 40 ${}^{\circ }$C) and total fermentation time of 60 h, where Table 3 shows the final value at the end of the simulation. It is evident from Figure 1 that cell mass growth was, initially, slower compared to ethanol at high temperatures. The 20 ${}^{\circ }$C model achieved a higher final ethanol concentration even though it had a slow start compared to the higher temperatures. High temperatures had a massive positive effect hence accelerating the production of ethanol, which caused the acceleration of cell decay. For example, for the 40 ${}^{\circ }$C model, the ethanol and cell mass concentrations increased faster; thus, the cell growth started to be repressed, and final ethanol concentration was compromised. Nonetheless, ethanol concentration increased with prolonged periods of fermentation even though the metabolic process of yeast producing ethanol has diminished.

### 3.2. Model Validation

Other fermentation models using *Saccharomyces cerevisiae* were compared with the studied computational model [11,12]. These models were selected since they used Simultaneous Saccharification and Fermentation as the fermentation method (SSF) and had similar temperature ranges compared to the computer model analyzed. However, it was not expected that the results of different fermentation process would be identical to the proposed model since they studied different yeast strains or parameters. Additionally, they did not state if the temperature was controlled or corrected during the fermentation. More details and differences about these fermentation processes can be observed in Table 4. The simulations used the same characteristics for temperature, time, glucose concentration, and ethanol inhibition degree (n) of the experimental data. If a parameter were not explicit in the compared study, it would be manually changed to fit the best final ethanol concentration value.

**Figure 2:**
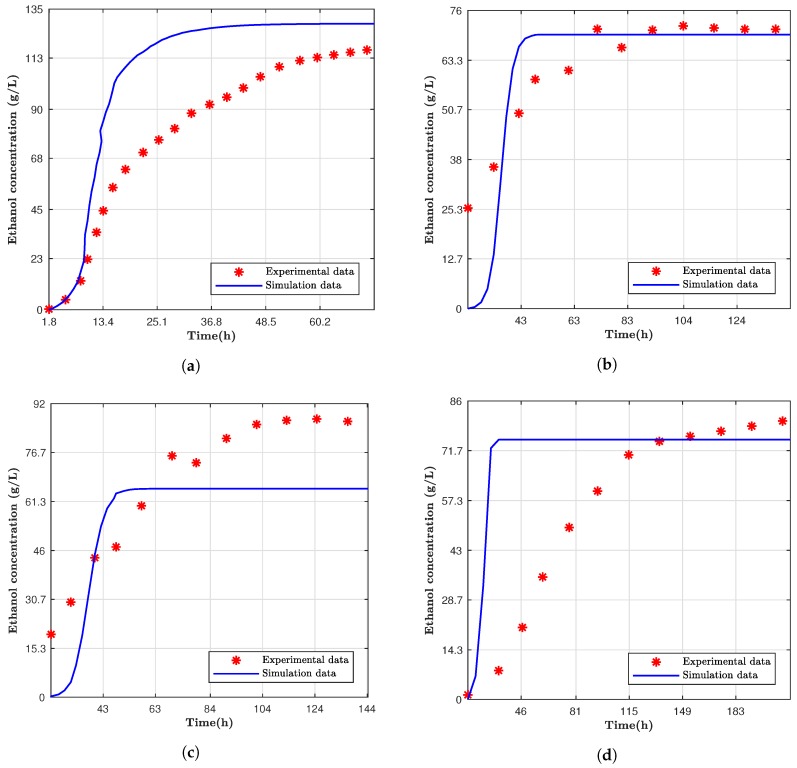
Simulation data compared to experimental data from (**a**) Devantier et al. [12]; (**b**) Nguyen et al. [11] with carbon starvation at 37 ${}^{\circ }$C and (**c**) 30 ${}^{\circ }$C, and (**d**) both without carbon starvation at 37 ${}^{\circ }$C.

Overall, the computer model reached a constant ethanol concentration faster than the experimental data, as can be observed in Figure 2; however, the final production values were proximate to the models. Devantier et al. had a similar final ethanol concentration; however, there were significant differences over time, as seen from Figure 2a. For the Nguyen et al. results, the trend over time of the ethanol production was very different. However, the concentration of the alcohol at the end was very close for fermentation cases where the temperature was 37 ${}^{\circ }$C. Table 5 shows the difference of the final values on the ethanol production, and, as previously described, it can be observed that Nguyen et al., for the two fermentation data at 37 ${}^{\circ }$C, had the smallest differences.

### 3.3. Sensitivity Analysis

A parametric sensitivity analysis was performed to assess the effect of independent variables such as the time (h), yeast concentration (g/L), and the temperature (${}^{\circ }$C) on the total production and production rate of ethanol. The predictive analysis was implemented based on the Gaussian Process. The model showed a strong correlation between the actual and predicted ethanol production and production rate of ethanol, which indicated that the model was valuable in its prediction approximation. For the total ethanol production, the model results suggest that there are three main effects. An increase in yeast concentration (main effect 0.833, Table 6) leads to more production than when having increasing temperature and time. The temperature (20 ${}^{\circ }$C to 40 ${}^{\circ }$C) had consistent ethanol production and the prolonged period (12 h to 90 h) had a positive relationship with the ethanol production (the longer production time was, the more ethanol was produced), while the yeast concentration was increased. Moreover, it was determined that the yeast concentration has the highest sensitivity (Table 6), which evidently shows that the yeast explained a significant variation in ethanol production.

The rate at which the ethanol was produced was proportional to the amount of yeast concentration added. Just as above, the Gaussian Process model indicates that the temperature consistently (as observed from marginal plots: flat relationship) contributed to the production rate of ethanol, while the yeast concentration kept on increasing. On the other hand, the prolongation period had a negative quadratic relationship on the ethanol production rate. As the ethanol production was allowed to run longer, the production rate decreased further and fast approached the zero mark regardless of the higher temperature and yeast concentration. In addition, the time had the highest sensitivity (Table 6), which evidently shows that this component explained a significant variation in reduced ethanol production rate (even though more time did not contribute any significantly to final ethanol production). If anything, the model showed that long times are not necessary. However, more simulation studies ought to be conducted to optimize the ethanol production rate period and energy consumption. In summary, the increase in yeast concentration acts as the primary catalyst and energy that leads to more production of ethanol, more specifically at the transient stage and stabilizes with time and temperature until little or no ethanol is produced. Moreover, the yeast concentration can only be increased so much, until there is no resultant ethanol production in the batch. The interaction effects on ethanol production and production rate were observed to be less significant in the model. According to the simulation model, the yeast concentration is critical to the production of ethanol.

### 3.4. Variable Temperature Modeling

Since the temperature has a crucial influence on ethanol production rate, the effect of changing this parameter during fermentation was also studied. As observed from Figure 1, the ethanol production at 40 ${}^{\circ }$C was more accelerated, but the maximum total ethanol concentration was gained at 20 ${}^{\circ }$C. Thus, in this study, a fermentation model was designed where the temperature was controlled to change from 40 ${}^{\circ }$C to 20 ${}^{\circ }$C once within 24 h and then within 60 h. This controlled temperature model adjusted the temperature of the fermentation with time to start at 40 ${}^{\circ }$C and exponentially decreased with time until 20 ${}^{\circ }$C at the end of the process. The intention was to put the yeast cells in an accelerated growth mode but reduce the temperature to minimize stress and, potentially, lessen cell decay.

**Figure 3:**
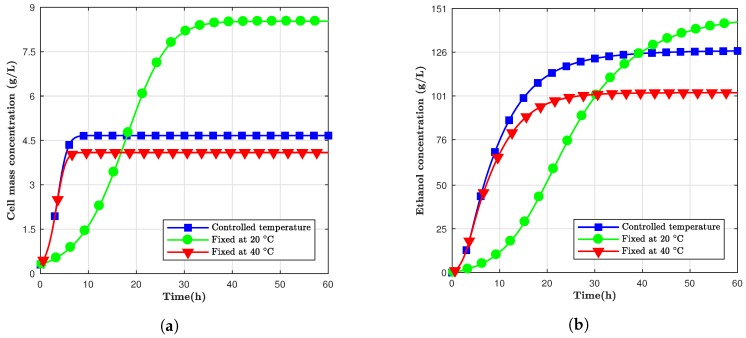
The concentration of (**a**) cell mass and (**b**) ethanol for simulations at fixed and controlled temperatures in a 60 h window.

Figure 3 shows three different scenarios. From Figure 3a, it can be observed that cell mass concentration grew faster than the cells kept at 20 ${}^{\circ }$C; however, it did not reach the same pinnacle at the end of the sixty hours. Figure 3b shows the total ethanol mass versus time for 60 h and for fermentation at 20 ${}^{\circ }$C, 40 ${}^{\circ }$C and changing from 40 ${}^{\circ }$C to 20 ${}^{\circ }$C for the same period. The controlled temperature simulation had the highest yield until, approximately, 40 h past the start of the fermentation process, but the simulation kept at 20 ${}^{\circ }$C ended up having the highest concentration for both ethanol and cell mass. Therefore, depending on the time window, the controlling temperature could be more advantageous than maintaining it constantly.

**Figure 4:**
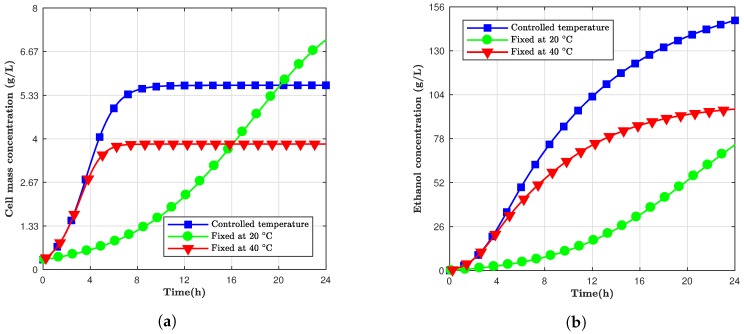
The concentration of (**a**) cell mass and (**b**) ethanol for simulations at fixed and controlled temperatures in a 24 h window.

Considering a fermentation period of 24 h, the same controlled temperature simulation was designed. The results can be observed in Figure 4, where the final ethanol concentration for the controlled temperature was 148.0 g/L. Even though the cell concentration for 20 ${}^{\circ }$C was still higher after 24 h, the ethanol concentration for the controlled model was 35.5% and 49.7% higher compared to the 40 ${}^{\circ }$C and 20 ${}^{\circ }$C models. This modified model took advantage of the high initial production rate at elevated temperatures and steady cell mass concentration at inferior temperatures. Despite the fact that the cell production stopped, the ethanol production continued since not all yeast cells were inactive. However, depending on the time window to be utilized, using a variable temperature approach could be less advantageous than just keeping it constant.

Table 7 shows the values for cell mass and ethanol concentration at the end of the simulation. After 60 h, the ethanol concentration for the simulation fixed at 20 ${}^{\circ }$C was 11.6% higher compared to the variable. It can be observed that the 24-h simulation was 14.4% higher compared to the 60-h one. The 24-h model had a faster temperature pace; thus, the desirable effects of controlling the temperature influenced the process in a more immediate way for the shorter time window model.

The controlled temperature model presented higher ethanol yields after 24 h compared to the fixed temperature models, but none of the simulations reached a constant value. In other words, the cells still needed more time to achieve the maximum concentration possible. The controlled temperature modeling could be applied depending on the time to be subjected to the fermentation process. At the same time, this suggested model could help monitor or accelerate the process to a desirable ethanol concentration.

## 4. Conclusions

The nonlinear system studied was able to model fermentations processes at different fixed temperatures using the *S. cerevisiae*. It showed how elevated temperatures had faster cell mass growth but a shorter final ethanol concentration. There was an indication that the final ethanol concentration was more sensible to the initial glucose amount compared to temperature and time. This study emphasized the use of a controlled variable temperature model over a fixed temperature to maximize ethanol yields. However, further investigations are needed to implement this presented strategy in industrial processes, and the economic costs demanded for this purpose. This approach could benefit ethanol producers to maximize production, as a monitoring tool of the process, as well as an educational tool for the instruction of this essential biological system.

## Figures and Tables

**Table 1:** List of thermodynamics characteristics for the kinetic parameters [10].

Kinetic Parameter	A (h${}^{-1}$, L g${}^{-1}$ h${}^{-1}$) *	E (cal/mol)
$\mu_{0}$	$8.84\times 10^{8}$	$1.30\times 10^{4}$
$k^{\prime }$	$7.12\times 10^{14}$	$2.34\times 10^{4}$
$\beta_{0}$	$1.48\times 10^{7}$	$9.76\times 10^{3}$
$k^{\prime \prime }$	$1.76\times 10^{4}$	$1.00\times 10^{4}$

* h${}^{-1}$ for $\mu_{0}$ and $\beta_{0}$; L g${}^{-1}$ h${}^{-1}$ for $k^{\prime }$ and $k^{\prime \prime }$.

**Table 2:** Range of input parameters for LHS design.

Parameter	Lower Limit	Higher Limit
Yeast concentration (g/L)	0	10
Temperature (${}^{\circ }$C)	20	40
Time (h)	12	90

**Table 3:** Final values after a 60 h simulation for cell mass concentration and ethanol concentration.

Temperatures (${}^{\circ }$C)	Cell Mass Concentration (g/L)	Ethanol Concentration (g/L)
20	8.72	145.8
26	6.68	131.5
33	5.36	119.0
40	4.53	110.1

**Table 4:** Comparison between experimental and computational data.

Parameters	Nanba et al. [10]	Devatier et al. [12]	Nguyen et al. [11]
*S. cerevisiae* strain used	HUT 7170	Fermiol HA and Red Start Ethanol Red	$D_{5}$A
Main Feedstock	Corn	Corn	Corn Stover
Fermentation Type	Batch and SSF	Very High Gravity bath mashes and SSF	Batch and SSF
Initial Temperature (${}^{\circ }$C)	Range of 15 to 40 in steps of 5	32	30 and 37
Fermentation time (h)	Until the ethanol production was constant	72	144

**Table 5:** Final ethanol concentration values (g/L) for experimental and simulated data.

Authors	Experimental Value (g/L)	Simulation Value (g/L)	Difference
Devantier et al.	117.4	128.5	−8.64%
Nguyen et al. (b)	71.6	69.8	1.90%
Nguyen et al. (c)	86.3	65.4	32.0%
Nguyen et al. (d)	81.0	74.8	8.20%

(b) with carbon starvation at 37 ${}^{\circ }$C, (c) with carbon starvation at 30 ${}^{\circ }$C, (d) without carbon starvation at 37 ${}^{\circ }$C.

**Table 6:** Sensitivity analysis of the independent variables on total ethanol production and ethanol production rate.

Input Variables	Total Sensitivity		Main Effect	
	Total Production	Production Rate	Total Production	Production Rate
Temperature (${}^{\circ }$C)	0.0289	0.0129	0.0037	0.0004
Yeast concentration (g/L)	0.8568	0.2910	0.8328	0.2124
Time (h)	0.0805	0.7542	0.0548	0.6743

**Table 7:** Final cell mass and ethanol concentration after 24 and 60 h simulations for fixed and controlled temperatures.

Temperature (${}^{\circ }$C)	Cell Mass (g/L)		Ethanol (g/L)	
	24 h	60 h	24 h	60 h
20	7.03	8.53	74.4	143.29
40	3.84	4.09	95.5	102.83
Controlled	5.64	4.66	148.0	126.7
